# Effects of schema on the relationship between post-encoding brain connectivity and subsequent durable memory

**DOI:** 10.1038/s41598-023-34822-4

**Published:** 2023-05-30

**Authors:** Dingrong Guo, Gang Chen, Jiongjiong Yang

**Affiliations:** 1 School of Psychological and Cognitive Sciences and Beijing Key Laboratory of Behaviour and Mental Health, Peking University, Beijing, 100871 People’s Republic of China; 2 Scientific and Statistical Computing Core, National Institute of Mental Health, Bethesda, MD USA

**Keywords:** Neuroscience, Cognitive neuroscience, Learning and memory

## Abstract

Schemas can facilitate memory consolidation. Studies have suggested that interactions between the hippocampus and the ventromedial prefrontal cortex (vmPFC) are important for schema-related memory consolidation. However, in humans, how schema accelerates the consolidation of new information and relates to durable memory remains unclear. To address these knowledge gaps, we used a human analogue of the rodent spatial schema task and resting-state fMRI to investigate how post-encoding brain networks can predict long-term memory performance in different schema conditions. After participants were trained to obtain schema-consistent or schema-inconsistent object-location associations, they learned new object-location associations. The new associations were tested after the post-encoding rest in the scanner and 24 h later outside the scanner. The Bayesian multilevel modelling was applied to analyse the post-encoding brain networks. The results showed that during the post-encoding, stronger vmPFC- anterior hippocampal connectivity was associated with durable memory in the schema-consistent condition, whereas stronger object-selective lateral occipital cortex (LOC)-ventromedial prefrontal connectivity and weaker connectivity inside the default mode network were associated with durable memory in the schema inconsistent condition. In addition, stronger LOC-anterior hippocampal connectivity was associated with memory in both schema conditions. These results shed light on how schemas reconfigure early brain networks, especially the prefrontal-hippocampal and stimuli-relevant cortical networks and influence long-term memory performance.

## Introduction

New memories are not stored on a tabula rasa: prior knowledge or schemas influence how our memories are formed. A schema is a knowledge structure that guides encoding, consolidation and retrieval of information that can be related to prior knowledge (the schema)^1^. Although the definition of a schema has varied in the literature (see the review^2^), most theories place the ventromedial prefrontal cortex (vmPFC), hippocampus and the interaction between them at the centre of schema-related memory processes^3–5^. To date, most research on schemas has focused on the roles of vmPFC and the hippocampus regarding how schemas drive encoding and retrieval (see reviews^1,6^). However, little research has been devoted to how hippocampal and frontal activity during post-encoding consolidation relates to subsequent memory^7–9^.

Post-encoding brain activities have been proposed to mediate long-term memory stabilisation and transformation after memories are encoded^10^. Human functional neuroimaging studies have revealed that brain connectivity during post-encoding rest can be correlated with subsequent memory performance^11–16^, suggesting that post-encoding connectivity may reflect early memory consolidation processes by which newly-encoded memories become more stable.

Schema-related studies have also explored the effect of schema on memory consolidation by examining the relationship between post-encoding vmPFC–hippocampal connectivity and memory performance^8,9,17,18^. For example, a study^18^ addressed this question by associating houses with familiar or unfamiliar faces and found that schemas (prior knowledge) increase functional connectivity between the hippocampus and vmPFC. However, they did not identify a relationship between this connectivity and immediate memory performance. Using a delayed memory test (72-h after encoding), Audrain and McAndrews found that the hippocampal–vmPFC connectivity during post-encoding predicted durable memory^9^. Recent evidence from animal and human neuroimaging studies has shown that schema advantage seems to be more pronounced in the delayed retrieval (e.g., a larger behavioural schema effect) and induces faster hippocampal activity decline^19–22^. Some theories have also proposed that schema may tag early neural changes immediately after encoding, and those changes may influence later memory consolidation and then improve long-term memory performance^23^. Therefore, the post-encoding vmPFC–hippocampal connectivity is more likely to predict long-term memory performance.

In contrast to the intraexperimentally learned spatial schema in rodent studies^24,25^ and following human studies focusing on encoding and retrieval^21,22,26–28^, in previous human studies focusing on memory consolidation, real-world schemas, such as congruent films^8^, famous faces^18^ and congruent object-scene pairs^9^ have been manipulated as schemas. Because schemas are related to known information, long-existing schemas may induce more complex brain networks, such as semantic networks, which may confound the results of ‘early consolidation’. How a newly built schema without overlapping to long-existing knowledge, accelerates the consolidation of information remains unclear^29^. Solving this problem will require investigating the relationship using an intraexperimental paradigm (i.e., a human analogue of the rodent spatial task), which can also bridge the gap between human and rodent studies.

In the present study, resting scans were performed on participants after the object-location encoding tasks^22^ in experimentally trained schema-consistent (schema-C) and schema-inconsistent (schema-IC) conditions. Object–location memory was then tested immediately and 24 h later. We sought to directly examine how post-encoding connectivity can predict long-term memory performance, especially connectivity between the vmPFC and the hippocampus. Recent work suggests that information may be represented along the longitudinal axis of the hippocampus by detailed representations in the posterior hippocampus and generalised representations in the anterior hippocampus^4^. The results of our previous study also showed the dissociation of the vmPFC–anterior hippocampus (aHPC) and vmPFC–posterior hippocampus (pHPC) connectivity in schema-related and schema-unrelated memories^22^. Therefore, we included vmPFC, aHPC and pHPC as our a priori regions of interest (ROIs). Specifically, the anterior hippocampus represents more global features such as abstract context^4,30^. In a human imaging study, Tompary and Davachi (2017) found that vmPFC–aHPC connectivity was associated with remote memory representation. Furthermore, Audrain and McAndrews^9^ showed that the post-encoding vmPFC–aHPC connectivity rather than the vmPFC–posterior hippocampus (pHPC) connectivity was related to long-term schema-congruent memory. Therefore, our first hypothesis was that vmPFC–hippocampal connectivity, especially vmPFC–aHPC connectivity, would be related to long-term memory in the schema-C condition.

In addition, given that the schema involved visual objects, the object-selective lateral occipital cortex (LOC) was added as our ROI. This region plays an important role in object-based schema formation^32^ and reactivation^33^, as well as in the reinstatement of prior mnemonic information^34^. The post-encoding interactions between LOC and the hippocampus are also related to later memory^15,35–40^. Therefore, the connectivity between LOC and hippocampus could be also related to later memory, especially in the schema condition-IC condition that needs more visual details.

Finally, in a larger network, we further included two other key regions, the angular gyrus (AG) and posterior cingulate cortex (PCC), as our ROIs, which are regarded as central to episodic memory networks^41–43^. Specifically, in schema-related processes, these regions may be engaged in integrating contextual information and rule-based formation^32,44,45^. Moreover, as we measured resting-state connectivity, those two key regions and vmPFC were also considered core hubs of the default mode network (DMN, which deactivates when any task is performed and activates during rest)^46,47^. It has been proposed that DMN is involved in generating and retrieving schemas to dynamically interpret an external situation^48^. A recent study also suggests that schema memory is supported by a specific reconfiguration of the DMN^27^. Therefore, including the AG and PCC in our analyses allowed us to specify whether the effect of schema on relationships between post-encoding and memory performance was specific to vmPFC–hippocampal connectivity or whether this was a global effect related to networks in episodic memory or DMN.

## Material and methods

### participants

A total of 23 participants (13 female; mean age = 21.56 years, SD = 2.58) were used in this study, including 19 participants from the previous study^22^ and 4 (a counterbalanced cell) newly recruited participants. All participants were from the Peking University community and were compensated for their participation. All were native Chinese speakers and gave written informed consent in accordance with procedures and protocols approved by the department Review Board of Peking University. All methods were performed in accordance with the relevant guidelines and regulations.

### Materials and procedures

**Figure 1:**
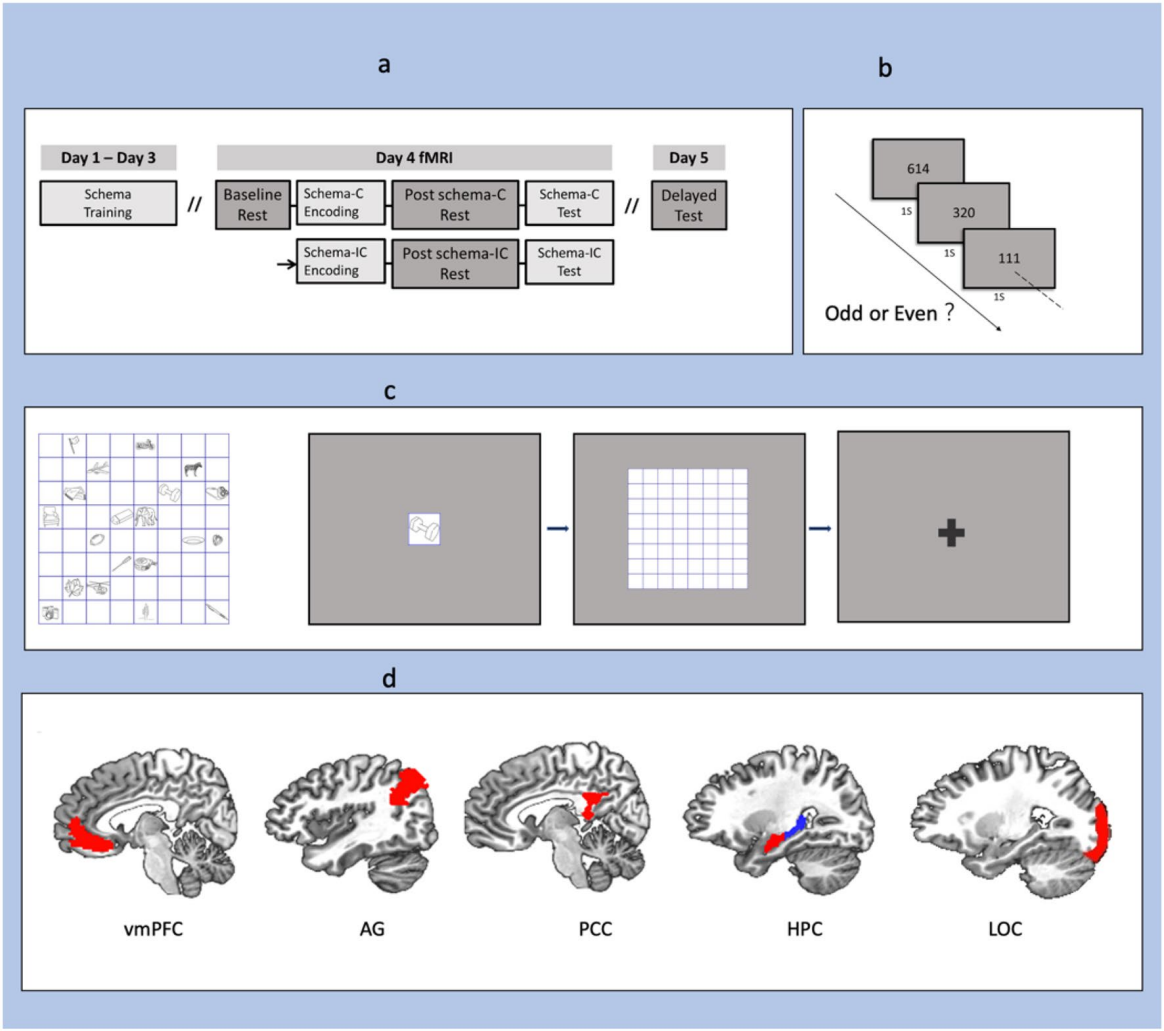
Experimental procedure and tasks, regions of interest. (**a**) Overview of the procedure, including three resting scans: baseline rest, post-schema-C rest and post-schema-IC rest. The order of two schema conditions (schema-C, schema-IC) was counterbalanced across participants. (**b**) Odd/even task during resting scans. In the task, a random three-digit number was presented on the screen every second. Participants were asked to press button 1/2 when the number was odd/even (counterbalanced across the participants). (**c**) Example of PAs and a typical trial in the memory test. At the start of learning each grid, the grid with all the PAs was presented for 90 s. After that, during each trial, an object cue was presented for 1 s, and the participants were asked to recall and choose the corresponding location in 3 s. (**D**) 12 brain ROIs (only showing left hemisphere): vmPFC, angular gyrus (AG), posterior cingulate cortex (PCC), anterior and posterior hippocampus (HPC, red for anterior and blue for posterior) and lateral occipital cortex (LOC).

The object–location pair associations (PAs) were used to manipulate schema in this study. There are two schema-C grids and two schema-IC grids, the PAs were located on either the schema-C or schema-IC grids. The details were reported in a separate study focused on memory retrieval^22^, and the following is a summary of the materials and procedures. There were two sessions: a training session and a new learning session (Fig. 1a). The training session was performed during days 1–3. In this session, the participants were trained to learn four grids of 20 object–location PAs. During learning, each grid was presented on the screen for 90 s at the start and the participants were told to remember the locations of all the objects, then the PAs in the grid were tested with feedback successively. The new learning session was performed on days 4 and 5. During this session, the participants learned 12 new PAs and were tested on these new PAs and the 8 PAs learned in the training session within each 8 × 8 grid. The learning process was like that in the training session. The tests were immediate (day 4) and delayed (day 5) object–cue recall tests, during which the grids with all PAs were not shown at the start and the feedback was no longer provided. Only on day 4, the participants performed the tasks in the fMRI scanner. Experimental analyses focused on the data of resting scans.

The schema-C and schema-IC grids were different regarding the consistency of the trained PAs across the training days. On a schema-C grid, the 20 trained PAs remained unchanged and were consistent from day 1 to day 3. On a schema-IC grid, the objects and their possible locations of the 20 trained PAs were fixed, but the combinations between them changed across the training days, although they were consistent within the same day. Therefore, before the new learning session (inside the scanner) on day 4, the trained PAs on the schema-C grid had been overlearned in the training session, and a stable spatial associative schema of object–location PAs could have been established. However, the combinations of the trained PAs on the schema–IC grid were completely new, and no such stable schema had been established. In the new learning session, there were 16 trained PAs (8 on each grid) and 24 newly learned PAs (12 on each grid) in the two grids of each schema condition.

For the fMRI data, three resting scans were performed: a baseline resting scan before all the tasks, a post-schema-C scan following the schema-C encoding task and a post-schema-IC scan following the schema-IC encoding task (Fig. 1a). Each resting scan lasted 5 min 12 s, during which participants were required to perform an odd/even task (Fig. 1b) to prevent intentional rehearsal^14^. It could serve as an active resting scan in memory studies since memory-related brain network has been shown to be uncompromised in such cognitive subtractions^49^. In the odd/even task, a random three-digit number was presented on the screen every second. Participants were asked to press button 1/2 when the number is odd/even (counterbalanced across the participants). The order of the two schema conditions was also counterbalanced across the participants. There are two memory tests for each schema condition: an immediate test following each resting scan in the scanner and a 24-h delayed test outside the scanner. All the PAs of each grid learned and tested on day 4 were tested again on day 5 using the same procedure but in a different random order. During a memory test (Fig. 1c), for each trial, an object cue was randomly presented on the centre of the screen for 1 s. Then the participants moved the cursor and pressed the mouse’s left button to select the correct location within a response period of 3 s. After the selection, the grid turned grey, and the feedback was not presented. More procedure details can be found in our previous study^22^.

### fMRI data acquisition

A 3 T Siemens Prisma MRI scanner with a 20-channel head coil in the MRI Center at Peking University was used to acquire MRI images. In the structural MRI scan, T1-weighted high-resolution MRI volumes were obtained using a 3-dimensional magnetization-prepared rapid acquisition gradient echo (MPRAGE) sequence (FOV = 256 × 256 mm; matrix = 256 × 256; slice thickness = 1 mm, TE/ TR = 2.98/2530 ms, flip angle = 7°). High-resolution rest MRI image were obtained using a simultaneous multiband EPI sequence (FOV = 224 × 224 mm; matrix = 112 × 112, resolution = 2 × 2 × 2 mm, TE/ TR = 30/2000 ms, flip angle = 90°). Visual stimuli were presented using MATLAB 2014b (MathWorks, Natick, MA, USA) and elements of the Psycholotoolbox3^50^, back-projected to a screen, and viewed with a mirror mounted on the head coil. Responses were collected with an MRI-compatible mouse.

### fMRI analyses

The AFNI software package^51^ was used for the fMRI analyses. The first six volumes of the time series were removed to ensure that all remaining volumes were at steady-state magnetisation. We then performed outlier calculation (3dToutcount), despiking (3dDespike), volume registration (3dvolreg), and spatial smoothing with a 6-mm isotropic Gaussian filter (3dBlurToFWMH). Next, nuisance signal regression and band-pass filtering (0.01–0.10 Hz) were performed simultaneously, but only on volumes that survived motion censoring (< 0.3 mm between successive time points, based on the motion parameters) and excluded high-motion volumes. The regression step included 12 motion parameters, non-neuronal signals from eroded white matter and CSF masks and regressors for temporal filtering. Finally, we used AFNI’s ANATICOR function to eliminate local and global hardware artefacts. After pre-processing, the residual time series files, co-registered to the Talairach space, were used for all subsequent analyses.

We focused on six pre-defined ROIs: vmPFC, aHPC, pHPC, LOC, AG and PCC (Fig. 1d). We defined all the ROIs anatomically as in our previous study^22^. Bilateral hippocampus anatomical masks were created using AFNI’s FS_Desai_PM atlas, originally parcellated using FreeSurfer^52^. The anterior hippocampus was located at y >  − 21 in Talairach space and otherwise posterior^53^. The vmPFC anatomical mask was first defined using the Mackey vmPFC Atlas^54^, according to our previous study^22^ that shows the schema-related activation focuses in the 14-m subregion, the ROI here was further defined as the 14-m subregion in this mask. Masks of AG, PCC and LOC were all defined using AFNI’s CA_N27_ML atlas. We considered these brain regions in both hemispheres, so a total of 12 ROIs were included. For the three resting scans, the BOLD signal time series were extracted from each ROI by averaging across all voxels in that ROI. A correlation matrix was calculated for each subject using AFNI’s 3dNetCorr function. We then applied the Fisher z-transformation to each correlation coefficient and got the Fisher z-transformed correlation matrix.

For the group analysis, because our study was limited by sample size, we adopted the Bayesian multilevel modelling framework^55,56^. In this approach, the data from all effects of connectivity among ROIs are incorporated into a single multilevel model that integrates the hierarchical information across regions and subjects. Under the conventional mass univariate analysis, all the region pairs are assumed to have a uniform distribution instead of a more realistic Gaussian distribution. Even though the subsequent step of multiple testing adjustment may partly compensate for the relatedness among neighbouring regions, one would still have to pay the cost of heavy penalty as well as artificial dichotomization due to the uniform distribution assumption^57^. In contrast, the Bayesian multilevel modelling framework allows us to estimate the effects among regions as well as their uncertainty. In addition, full results can be presented: effects with strong evidence would be highlighted without hiding the rest. Under the conventional massively univariate framework through a general linear model, the effect at each region pair is estimated independently with no information shared across region pairs; hence, the multiple testing issue requires statistical evidence adjustment at the region pair level. In contrast, under the Bayesian multilevel framework, the data from all region pairs are included in a single hierarchical model, and the effect at each region pair is assessed through its posterior distribution. Adjustment for multiplicity is unnecessary because the inferences are drawn from the integrative model’s single, overall posterior distribution.

The Bayesian multilevel modelling framework was used for two kinds of analysis. The first was for brain-behaviour correlations, which examined the correlations between post-encoding brain connectivity and memory performance. Note that, although the analysis here was to determine how brain connectivity could predict the memory performance, to fit in a hierarchical model of all region pairs ^56^, behavioural performance was used as an explanatory variable for the Fisher z-transformed brain-network correlation matrix. In addition to the brain connectivity effects, the region effects were further estimated. The posterior distribution of a region effect represents how a particular region contributes to brain-behaviour relationships relative to other regions among all regions. The second analysis assessed the brain connectivity differences between two resting scans, which examined whether the trained schema could change the post-encoding brain connectivity. This analysis calculated a matrix of changes by subtracting the Fisher z-transformed correlation matrix of one resting scan from another. As the neural pattern was stronger in the participants who exhibited the schema effect^22^ , in all the analyses, we also examined 17 participants who demonstrated a schema effect (schema-C >  = schema-IC for memory performance on new PAs) in the delayed test. In addition, we added the order of the schema condition as a categorical factor in all the analyses, although it was counterbalanced across participants.

## Results

### Behavioural results

**Figure 2:**
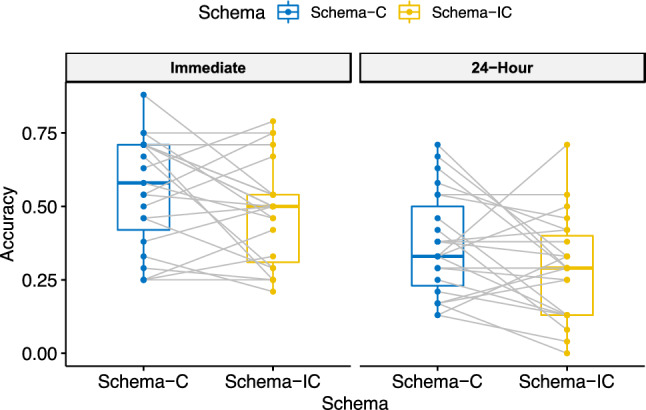
Behavioural results of all 23 participants. The box plots display the distribution of data based on the five-number summary: minimum, first quartile, median, third quartile, and maximum (from the bottom up); each point represents a participant and each grey line represent the schema-C to the schema-IC trend of the participant.

With four more participants plus 19 participants from the previous study, the behavioural results are similar to those reported in our previous study^22^. Again, only the trials in which participants chose the exact correct locations were considered as correct trials. In both the training and new learning sessions, memory accuracy was calculated as the proportion of correct trials for each condition (i.e., the number of correct trials out of the total number of trials). Briefly, the participants were trained during days 1–3. For the schema-related new learning, a 2 (schema: schema-C, schema-IC) ✕ 2 (time interval: immediate, 24-h) repeated measures ANOVA on newly learned PAs provided statistical evidence for the main effect of schema (0.46 ± 0.16 vs 0.38 ± 0.17, *F* (1, 22) = 4.528, *p* = 0.045, *η2* = 0.066, Fig. 2) as well as for the significant one-tailed schema effect for each time interval (immediate: 0.55 ± 0.18 vs 0.48 ± 0.17, *t* (1, 22) = 1.965, *p* = 0.032, d = 0.43; 24-h: 0.37 ± 0.18 vs 0.29 ± 0.16, *t* (1, 22) = 1.861, *p* = 0.039, d = 0.45). There was little evidence for the interaction between schema and time interval (*F* < 0.001, *p* = 1). Although the delayed memory did not show a larger schema effect in behavioural results, based on our hypothesis and our previous behavioural results with a larger sample showing greater delayed schema effect^22^, we focused on how the post-encoding brain connectivity could predict the memory performance after a one-day interval in the fMRI analysis, and then examined whether the connectivity also correlated with the immediate memory performance.

### fMRI results

All the fMRI results were analysed within a Bayesian framework. The full statistical evidence from Bayesian inferences is based on the full posterior distribution of an effect. Here, we report P + , the posterior probability of each effect being positive, as the extent of statistical evidence. Values of P + close to 1 indicate stronger evidence for the effect of interest (e.g., the correlation between brain connectivity and behaviour) being greater than zero. Values of P + close to 0 convey the extent of support for a negative effect. Therefore, the posterior probability that the effect was negative is (1–P +). Full results will be presented in the following part, although *P* +  >  = 0.85 or <  = 0.15 are marked as moderate extent of evidence.

#### Brain-behaviour associations

We assessed the brain–behaviour relationship for the connectivity matrix and delayed memory performance to test whether the post-encoding connectivity could predict the delayed memory performance. Under the schema-C condition, the connectivity between left and right AG showed some evidence (*P* +  = 0.12, see Figure S1a) for the negative effect of a connectivity-memory correlation, which means when their connectivity is lower, the memory performance is better in schema-C condition. Under the schema-IC condition, the inner connectivity network of vmPFC, AG and PCC also provided some evidence (*P* +  < 0.15, see Figure S1a) for the negative effect of connectivity–memory correlation, which means decoupling of this network could predict better memory in schema-IC condition. In addition, the connectivity between raHPC and rLOC also showed some evidence (*P* +  = 0.86, see Figure S1b) for the positive connectivity-memory correlation.

**Figure 3:**
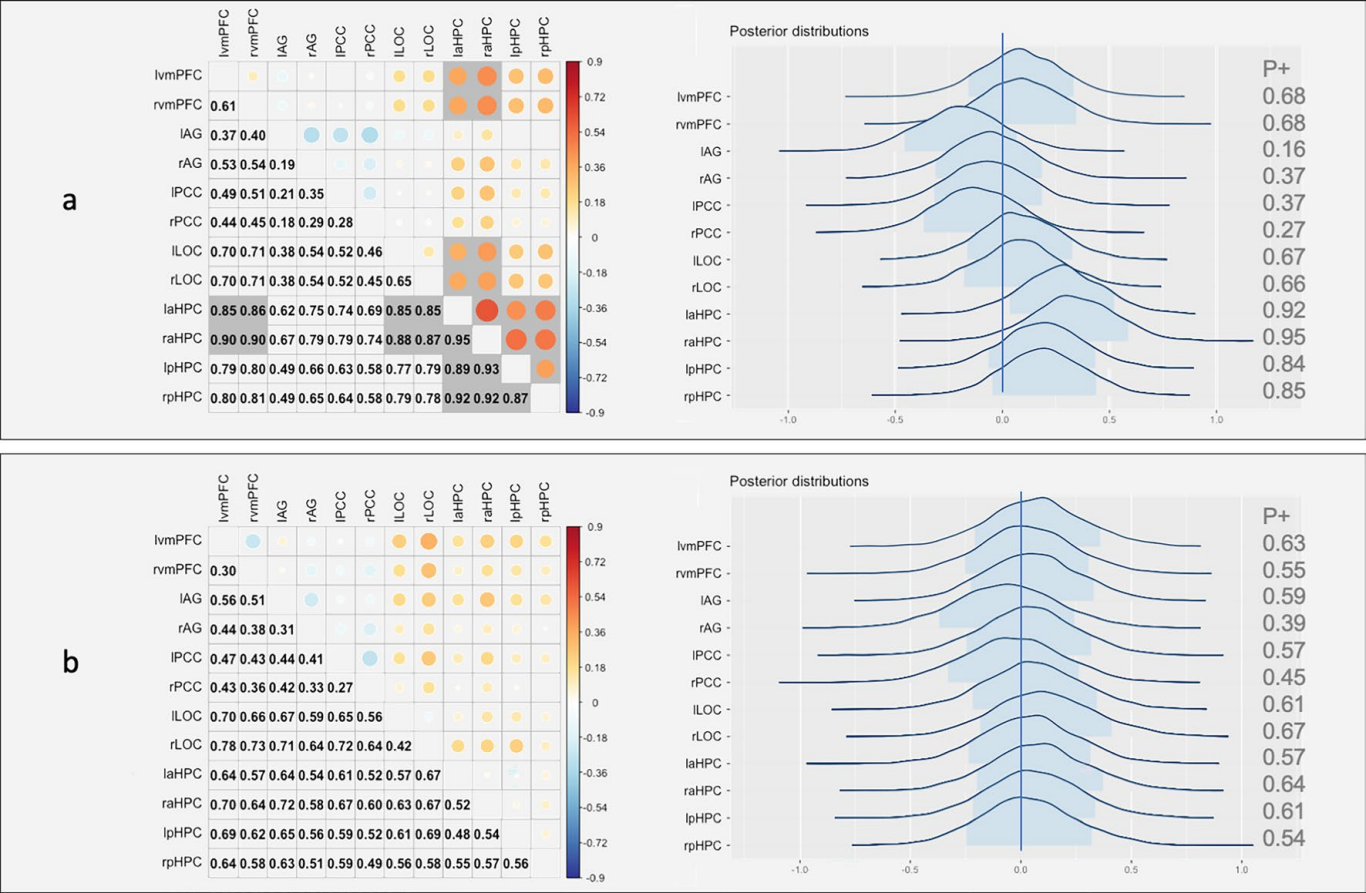
Association between brain connectivity and delay memory performance in schema-C condition for the 17 participants who demonstrated a schema effect. (**a**) Post-encoding and (**b**) baseline. On each of the two panels, the matrix shows region pair effects: the upper triangle illustrates the magnitude of the Fisher-transformed z-value, indicated by circle size and colour, and the lower triangle contains the *P* + value, *P* +  ≥ 0.85 or ≤ 0.15 are marked with grey colour. On the right of each panel are the posterior distributions for the region effects.

**Figure 4:**
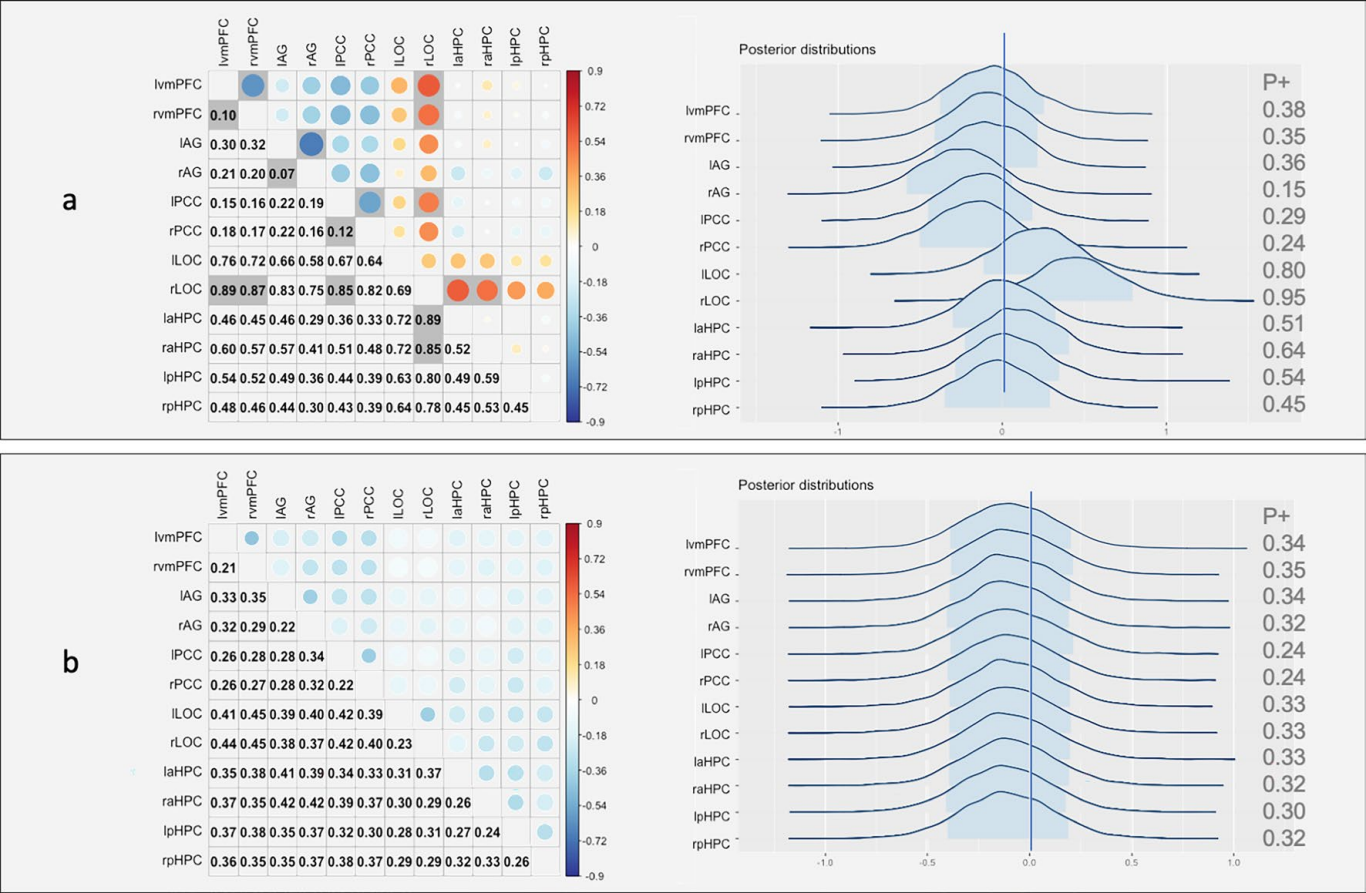
Association between brain connectivity and delayed memory performance in the schema-IC condition for the 17 participants who demonstrated a schema effect. (**a**) Post-encoding and (**b**) baseline. On each of the two panels, the matrix shows region pair effects: the upper triangle illustrates the magnitude of the Fisher-transformed z-value, indicated by circle size and colour, and the lower triangle contains the *P* + value, *P* +  ≥ 0.85 or ≤ 0.15 are marked with grey colour. On the right of each panel are the posterior distributions for the region effects.

Then, the data of the participants who demonstrated schema effects were subject to further analysis. Post-schema-C connectivity that showed strong evidence for positive correlation with memory included prefrontal–hippocampal networks and LOC-anterior hippocampal connectivity (*P* +  > 0.85, Fig. 3a). Post-schema-IC connectivity showed similar results in all 23 participants, providing strong evidence that prefrontal–PCC–AG networks correlated negatively with memory (*P* +  < 0.15, see Fig. 4a), and vmPFC-LOC, aHPC-LOC connectivity correlated positively with memory (*P* +  > 0.85, see Fig. 4a). The region effects were further calculated. Results showed that under the schema-C condition, the anterior hippocampus-related connectivity had a crucial role relative to other regions for the brain-behavioural relationships among all regions (*P* +  = 0.92 and 0.95 for left and right aHPC respectively, Fig. 3a right); under the schema-IC condition, the LOC-related connectivity played the most important role in the relationship (*P* +  = 0.95 for the right LOC, Fig. 4a right).

Moreover, as a control, we performed the same analyses using the baseline rest. No baseline brain connectivity correlated with memory performance (Fig. 3b left and Fig. 4b left) and the region effects were not shown either (Fig. 3b right and Fig. 4b right), further indicating that the correlations and region effects were specific to the post-encoding connectivity. These results suggest that for the participants who demonstrated a schema effect, dissociated brain networks predicted the long-term memory performance in a different schema condition. In other words, higher connectivity between the anterior hippocampus and vmPFC was correlated with higher delayed memory in the schema-C condition. Under the schema-IC condition, post-encoding connectivity decoupling inside the networks of vmPFC, AG and PCC, and higher connectivity between stimuli-relevant region LOC and vmPFC predicted better memory performance. In addition, post-encoding connectivity between the anterior hippocampus and LOC was related to memory performance in both schema conditions.

Further, a new analysis was conducted to explore how differences in functional connections between two conditions could predict differences in memory performance. This was achieved through a similar model as above, where the memory difference between the two conditions in the delayed test was used as an explanatory variable for the matrix of changes. The changes were obtained by subtracting the Fisher z-transformed correlation matrix of post-schema-IC scan from the post-schema-C scan. The results indicated that the difference in connectivity related to the lateral occipital complex (LOC) between the two schema conditions may be crucial for the observed schema effect (Figure S2). This finding suggests that the schema effect may be linked to reduced processing of visual details.

**Figure 5:**
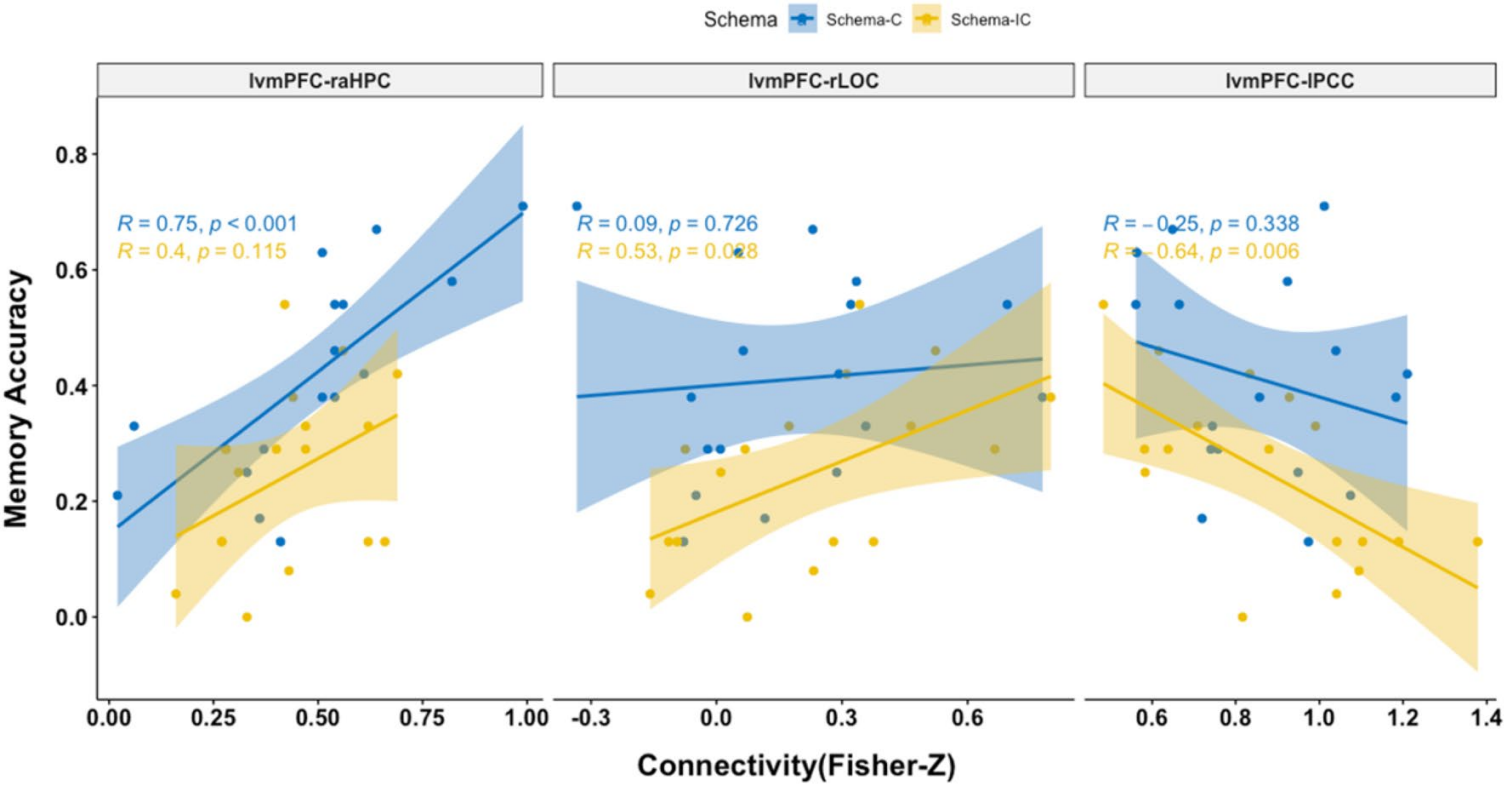
Scatterplots of the correlation between brain connectivity and memory performance in two schema conditions.

To further confirm and visualize the main results, we chose the connectivity between lvmPFC and three regions and calculated the corresponding Pearson correlation coefficients and examined the difference between coefficients of schema-C condition and schema-IC condition with a Z-test based on Monte Carlo stimulations^58,59^.As shown in the Fig. 5, the results were consistent with the results above from Bayesian multilevel modelling. Specifically, in the schema-C condition, post-encoding right anterior lvmPFC-raHPC connectivity positively correlated with delay memory performance (*R* = 0.75, *p* < 0.001), and the correlation was marginally stronger than the correlations in the schema-IC condition (*Z* = 1.62, *p* = 0.053). In the schema-IC condition, post-encoding lvmPFC-lPCC connectivity negatively (*R* =  − 0.64,* p* = 0.006) and lvmPFC-rLOC connectivity positively (*R* = 0.53,* p* = 0.028) correlated with delay memory performance respectively and, and the correlations were also stronger or marginally stronger than the correlations in the schema-C condition (lvmPFC-lPCC: Z = 1.58, *p* = 0.057; lvmPFC-rLOC: Z = 1.67, *p* = 0.048).

To clarify whether the post-encoding connectivity could also predict the immediate memory performance as demonstrated in previous studies ^8,18^, we did the same analyses regarding to the immediate memory performance. The results showed little evidence for the correlation between post-encoding brain connectivity in either schema condition and immediate memory performance (Figure S3).

#### Schema and brain connectivity changes

After examining the brain–behaviour correlations, we tested whether the schema condition modulated the post-encoding brain connectivity by calculating the matrix of changes for each schema condition by subtracting the baseline matrix from the post-encoding matrix. The modelling results showed little evidence that either schema condition changed the post-encoding brain connectivity (Figure S4a, b). We then directly compared the post-encoding brain connectivity between the two schema conditions and found little evidence for the difference between the two schema conditions for the post-encoding brain connectivity (Figure S4C).

All the analyses above were repeated with those 17 participants who demonstrated a schema effect. That is, there was little evidence that either schema condition changed the post-encoding brain connectivity (Figure S5a, b) or that there was a difference between the two schema conditions (Figure S5c). These results suggest that the schema that manipulated by object–location PAs could not induce substantial post-encoding connectivity changes significantly.

## Discussion

In the present study, we sought to investigate the effects of schema on the relationship between post-encoding brain connectivity and long-term memory performance. With a human analogue of the rodent spatial schema task and resting-state fMRI, the results showed that under the schema-C condition, post-encoding connectivity between the vmPFC and the anterior hippocampus predicted memory performance. Under the schema-IC condition, post-encoding connectivity decoupling inside the networks of vmPFC, AG and PCC, and connectivity between stimuli-relevant region LOC and vmPFC predicted memory performance. In addition, post-encoding connectivity between the anterior hippocampus and LOC was related to memory performance in both schema conditions. These results suggest that schema could modulate the relationship between brain networks post-encoding brain connectivity and subsequent durable memory. These findings offer novel insights into how schema tags early post-encoding brain networks and influences long-term memory performance.

With fMRI, it has been shown that the encoding of the overlapping information is associated with increased functional coupling between vmPFC and the hippocampus^60–62^ and is predictive of subsequent successful memory inference. Under the schema-C condition, previous results also showed schema-related vmPFC–aHPC connectivity in memory retrieval using the same paradigm^22^. The novel finding of our study was that there was a correlation between post-encoding vmPFC–aHPC connectivity and delayed memory performance. It suggests that the interaction may continue playing a role after learning and highlights the importance of offline prefrontal–hippocampal functional interaction in updating established neocortical memory traces via consolidation^3,63^. A previous study has shown that during the post-encoding period, there is enhanced hippocampal–vmPFC coupling after famous face encoding^18^, and our study further found that this coupling is related to durable memory. A recent study employing a pair association paradigm also showed that this coupling might be related to schema-congruent memory three days later^9^.These converging results suggest that schema may tag early neural changes with prefrontal–hippocampal (especially the anterior portion) connectivity, improving long-term memory performance^23^.

The observed correlation in the interaction between vmPFC and the anterior portion of the hippocampus is not surprising. Our previous studies with similar paradigm focusing on memory encoding and retrieval indicate that this region may carry the contextual (abstract) information of the newly formed schema^22,33^. The anterior hippocampus has also been associated with schema-based memory integration^60,61^ and contextual information detection^64^. One model also suggests that the anterior hippocampal signals carrying the contextual information are sent directly to the vmPFC, which then engages the appropriate rule and applies it to engage the context-appropriate representations^3,64,65^. Therefore, during the post-encoding rest, whether and how much the contextual or the schematic information from the posterior to the anterior hippocampus could continually send to the vmPFC may contribute to the schema-related consolidation and further influence the schema effect. This evidence suggests that vmPFC–aHPC connectivity plays a tagging role in this process and is vital in schema memory enhancement during early memory consolidation.

Under the schema-IC condition, the decoupling of vmPFC, AG and PCC networks predicted the memory performance. The vmPFC, AG and PCC are the main hubs of DMN. The decoupling of this system has been shown to directly associate with episodic memory^66^. During rest, medial temporal lobe structures, including the hippocampus, tend to be coupled with main DMN nodes. During episodic encoding, these structures are highly activated and are disconnected from other DMN nodes^67^ when arbitrary associations are encoded, while the connectivity of this network is increased when the encoding involves prior knowledge^68^ and their subnetworks may hold specific event information that is encoded in the vmPFC-hippocampal memory traces^69^ . The DMN was also found to be involved in updating schemas when events violated expectations or were inconsistent with the established schema^48^. Therefore, decreased involvement of this network may indicate more memory-related processing and schema-based integration in the other networks, which is related to general or schema incongruent long-term memory.

Under both schema conditions, our results showed are a relationship between the LOC-anterior hippocampal connectivity and the memory performance. This is consistent with the role of this connection in general memory consolidation. Prior work has shown that representational regions spontaneously reactivate representations of recently encoded stimuli and their post-encoding interactions with the hippocampus are related to later memory^35–39^.A study also suggest a casual role of this interaction in episodic memory by post-encoding TMS to LOC^70^.

As one of the core hubs of memory networks, vmPFC is also thought to carry schema-level representations^71,72^ and to link the new information to its relevant schema^5,24,73^ and therefore is a hub of the schema-related brain network. A recent study suggests that a demand-specific reconfiguration of the vmPFC networks supports schema-related memory^27^. Evidence also indicates that flexibility in network connectivity is related to episodic memory^74^. Gilboa and Moscovitch^73^ proposed that the relevant schema for a situation is reinstated in vmPFC to prepare individuals for the type of information that they are likely to encounter. Then, the instantiated schema is instantiated to interact with the environment. This schema reinstatement has been found in the post-stimulus time frame^75^. However, in previous studies using similar spatial schema paradigm that focused on memory retrieval^22,26,27,76^, the activation of vmPFC didn’t differ in retrieving the newly PAs. We speculate that vmPFC might be more involved in schema detection and monitoring^5,21,77^, or in processing information that has been fully stabilized and assimilated in a schema network^22^. In the current study, as both the positive and negative relationships included vmPFC, the predictive networks (vmPFC-aHPC and vmPFC-LOC) in both schema conditions also included vmPFC, we propose that vmPFC may reinstate schematic information and then modulate how these brain networks are involved in schema-related and unrelated early memory consolidation via different brain networks. For instance, when we have a pre-existing framework of knowledge (a schema), the brain can consolidate new information quickly by using the connectivity between the vmPFC and the anterior hippocampus. The region effect in our results also showed that under the schema-C condition, the anterior hippocampus-related connevtivtiy had a crucial role in memory consolidation relative to other regions for the relationships among all regions. However, when we don't have a pre-existing schema, the brain needs more detailed information to consolidate the new memories. In this case, the connectivity between vmPFC and stimuli-relevant cortex LOC, as well as the decoupling of the default mode network (DMN), play a role in the consolidation process. The region effect also showed that under the schema-IC condition, the LOC-related connecvity played the most important role in memory consolidation.

Regarding memory consolidation, our results revealed a relationship between the post-encoding connectivity and delayed memory but not with the immediate memory. Most everyday memories are forgotten in daily life, only a few are retained for long-term storage. Other than the encoding, this selective process is related to memory consolidation, which can select some information to be retained but allow the rest to be forgotten. With a spatial schema, rats can acquire new associative memories based on single-trial learning, and these memories can become hippocampally independent within 48 h of memory consolidation^24,25^. These rodent studies have contributed to a revision of classical consolidation theory: the classical complementary memory system model consisting of two systems, a fast hippocampal memory system and a slow neocortical system, has now been extended to incorporate rapid consolidation in prefrontal cortical brain regions if new information is related to the schema^78^. With a newly learned spatial schema that is far removed from existing schemas (e.g., semantic networks), our study provides evidence that this consolidation is not only augmented in sleep^19,79^, but also sprouts and tags differently according to the schema in the immediate post-encoding phase. Nevertheless, we don't know if this spatial schema can also be applied and generalized to schema that humans typically use and encounter in daily life (e.g., in semantic memory). It is also interesting to think about whether the schema-IC condition applies to the real-world events. For instance, whether events do not adhere to a particular schema also interfere with each other, whether the role of schemas is to resolve interference across similar but unrelated memories, or whether schemas enhance memory through other means. Therefore, how the post-encoding brain activity and sleep interact to serve schema-related fast memory consolidation, and if different schemas work in the same way are topics worthy of further investigation.

## Limitations and future directions

Our study has several limitations that may suggest future research directions. First, we did not find that schema substantially changed brain connectivity. In our study, participants were trained for three days using a similar object–location task. They also performed similar practice trials before baseline scanning, which may have induced brain activity changes in the baseline rest and diminished the differences between schema conditions. Different from pure resting scans, the participants performed an add/even task in all the resting scans, which may confound the interpretation of resting state results and also diminish the different resting scans, although memory-related brain network has been shown to be uncompromised in such cognitive subtractions^49^. Another possibility is that the two post-schema rest scans interfered with each other depending on the counterbalancing order, such that for participants who completed schema-C encoding first, their schema-IC post-encoding rest scan may be influenced by both schema-IC and schema-C sessions, and vice versa. To address this question, it would be useful to conduct a between-subject analysis focusing only on the first post-encoding rest scans with a larger sample. This would allow us to analyze data that is "untainted" by the second encoding condition that follows. In addition, to prevent intentional rehearsal, participants performed odd/even in the resting scan, which may also diminish the differences between schema conditions. Second, we only found certain dissociated networks correlated with memory performance in participants who demonstrated a schema effect. In a previous study, although some post-encoding connectivity may not have been different between schema conditions, individual participants’ connectivity strength during the post-encoding rest could still predict associative memory performance^18^. As a control, we calculated whether brain connectivity during baseline resting was also correlated with memory performance. The results showed little evidence for brain–behaviour correlations, but the trends were similar to those of post-encoding. Previous studies have linked brain connectivity during rest with episodic memory ability^80,81^. Therefore, one possibility is that baseline rest in our study had been changed by training and experimental preparation (practice), and this baseline rest was related to training or personal traits, possibly further determined how the post-encoding network unfolded. This result also partially explains why some participants could not benefit from memory schema and why we observed only limited correlations among the participants who had schema effect.

Finally, it is important to acknowledge the limitations of our sample size for brain-behaviour correlations and the potential implications for generalizability of the findings. To address this limitation, we suggest that future studies with larger sample sizes and either the inclusion of all participants or a pre-registered plan to only include participants that exhibit a schema effect would be useful in confirming or extending our findings. Such studies could also address the role of individual differences (e.g., by separating participants that can benefit from that cannot benefit from schema, and through a developmental/ageing perspective) in schema-related memory processing. In addition, because all the grid line are visible, and every cell was occupied by an object picture, we counted correct trials as only the trials in which the correct location was chosen in this study. However, there is work showing that schema-supported memories are less precise than schema-irrelevant ones^82^. It would be useful to utilize a similar paradigm that the grid lines are invisible, and measure memory by more inclusive index, such as the trial error, which could be calculated as distance between the mouse click coordinate and the correct coordinate. It would be also interesting to correlate a more inclusive memory index with different post-encoding brain connectivity. Overall, we hope that our study provides some starting evidence for further research on the role of schema on post-encoding consolidation.

## Supplementary Information

Supplementary Information.

## Data Availability

The data that support the findings of this study are available from the corresponding author upon reasonable request.
