# Supplementary material for: Effects of schema on the relationship between post-encoding brain connectivity and subsequent durable memory
Source: Sci Rep. 2023 May 30;13:8736. doi: 10.1038/s41598-023-34822-4 (PMC10229577; doi:10.1038/s41598-023-34822-4)
Supplement: Supplementary file 1 — Supplementary Information. [file 41598_2023_34822_MOESM1_ESM.pdf]

Supplementary Information

for

Effects of schema on the relationship between post-encoding brain connectivity and subsequent durable memory

Dingrong Guo[1], Gang Chen[2] and Jiongjiong Yang[1]*

[1] *School of Psychological and Cognitive Sciences and Beijing Key Laboratory of Behaviour and Mental Health, Peking University, Beijing, PR China*

[2] *Scientific and Statistical Computing Core, National Institute of Mental Health, USA, Bethesda, MD, USA*

***Corresponding author:** Jiongjiong Yang, Ph.D., School of psychological and cognitive sciences, Peking University, Beijing 100871, P.R. China.

E-mail: yangjj@pku.edu.cn.

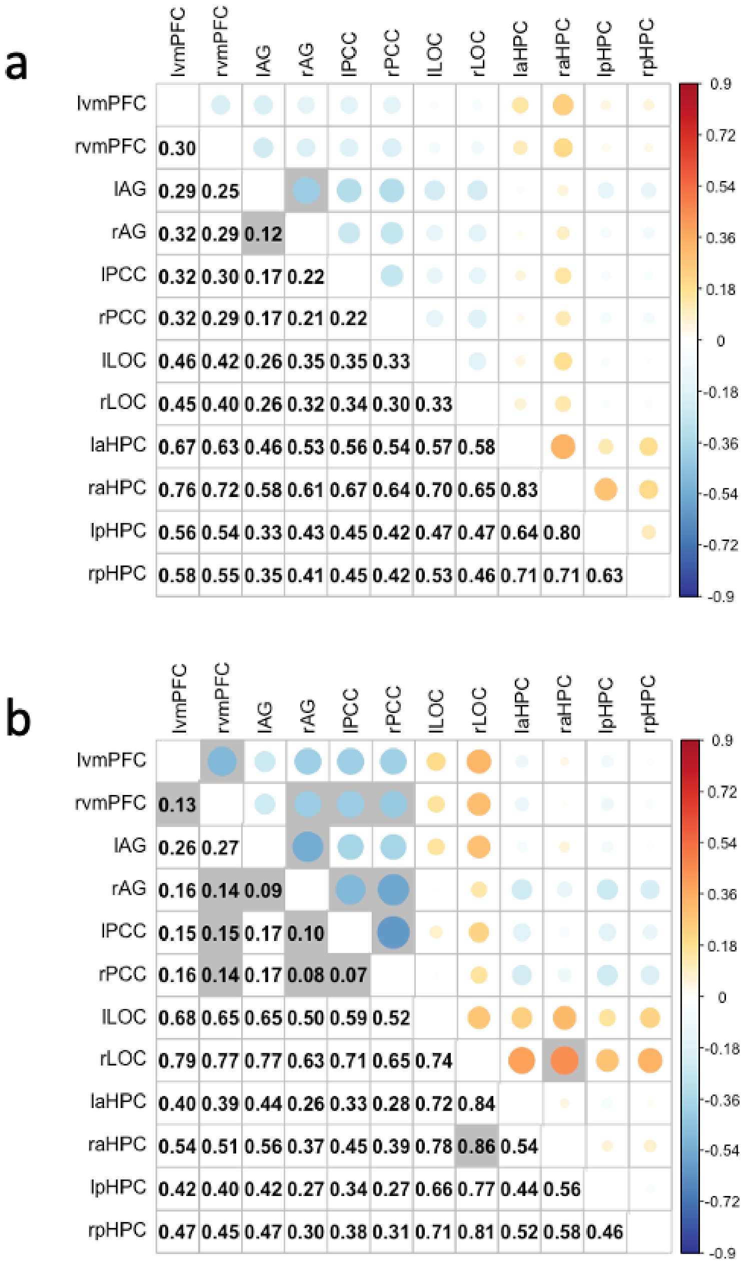

Figure S1. Association between brain connectivity and delayed memory performance in the schema-C condition (a) and schema-IC condition (b) for the 23 participants. On each of the two panels, the matrix shows region pair effects: the upper triangle illustrates the magnitude of the Fisher-transformed *z*-value, indicated by circle size and colour, and the lower triangle contains the P+ value， P+ ≥ 0.85 or ≤ 0.15 are marked with grey colour.

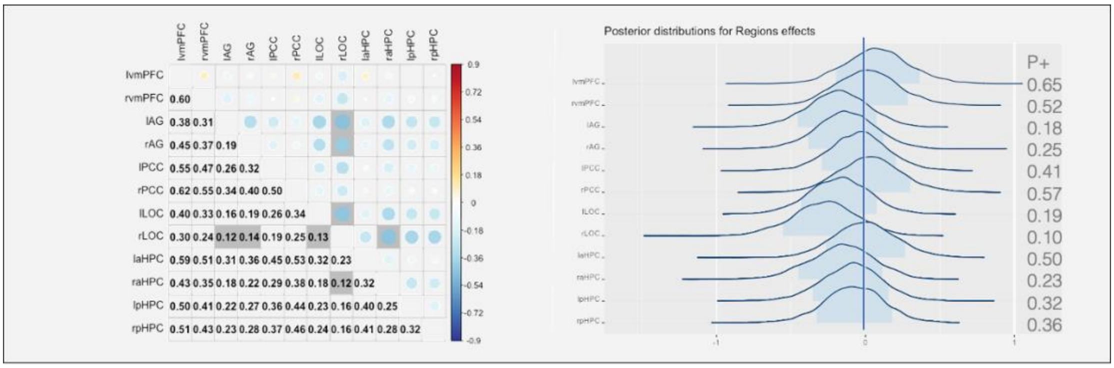

Figure S3. Association between brain connectivity and delayed memory performance for the 17 participants who demonstrated a schema effect. On the left panel, the matrix shows region pair effects: the upper triangle illustrates the magnitude of the Fisher-transformed z-value, indicated by circle size and colour, and the lower triangle contains the P+ value，P+ ⩾ 0.85 or ⩽ 0.15 are marked with grey colour. On he right panel are the posterior distributions for the region effects.

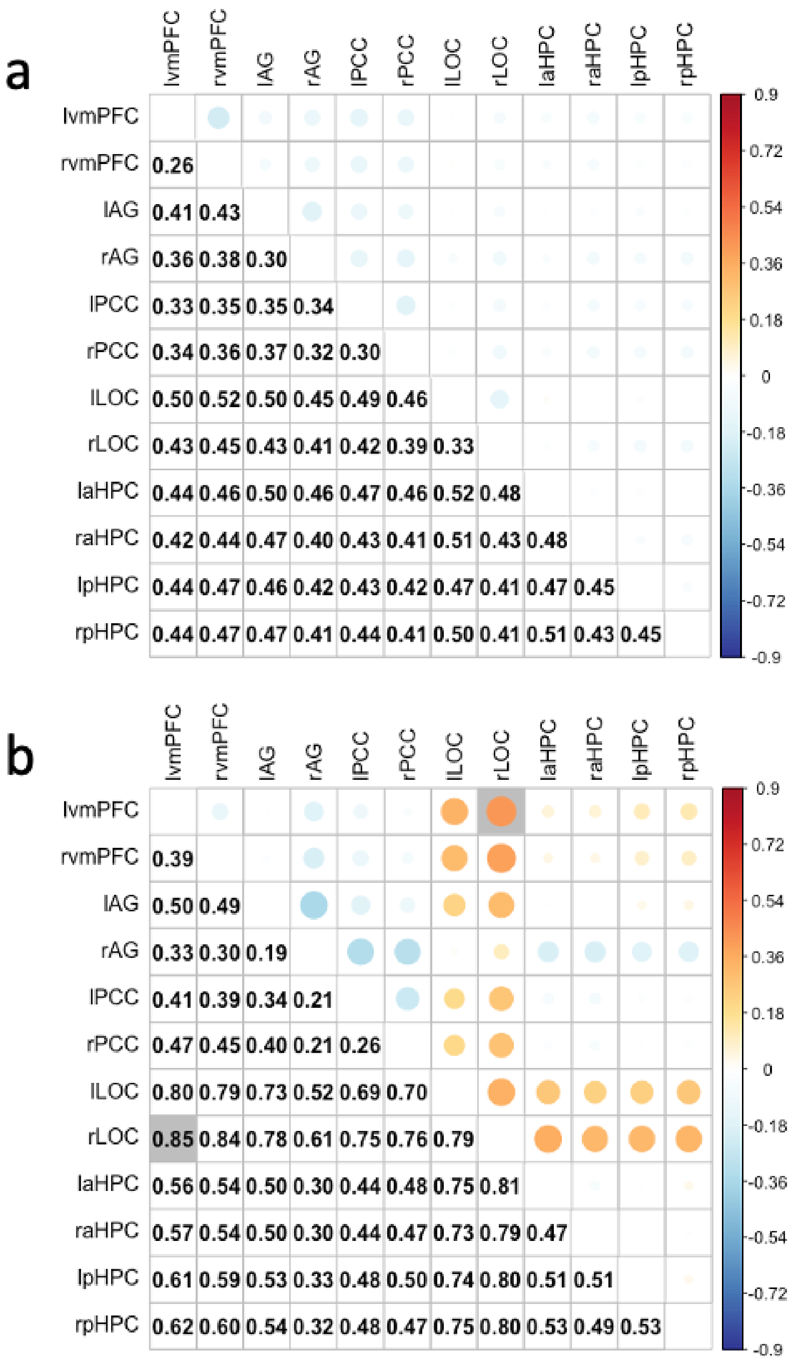

Figure S3. Association between post-encoding brain connectivity and immediate memory performance in all 23 participants. (a) Schema-C condition and (b) schema-IC condition. On each of the two panels, the matrix shows region pair effects: the upper triangle illustrates the magnitude of the Fisher-transformed z-value, indicated by circle size and colour, and the lower triangle contains the P+ value， P+ ≥ 0.85 or ≤ 0.15 are marked with grey colour.

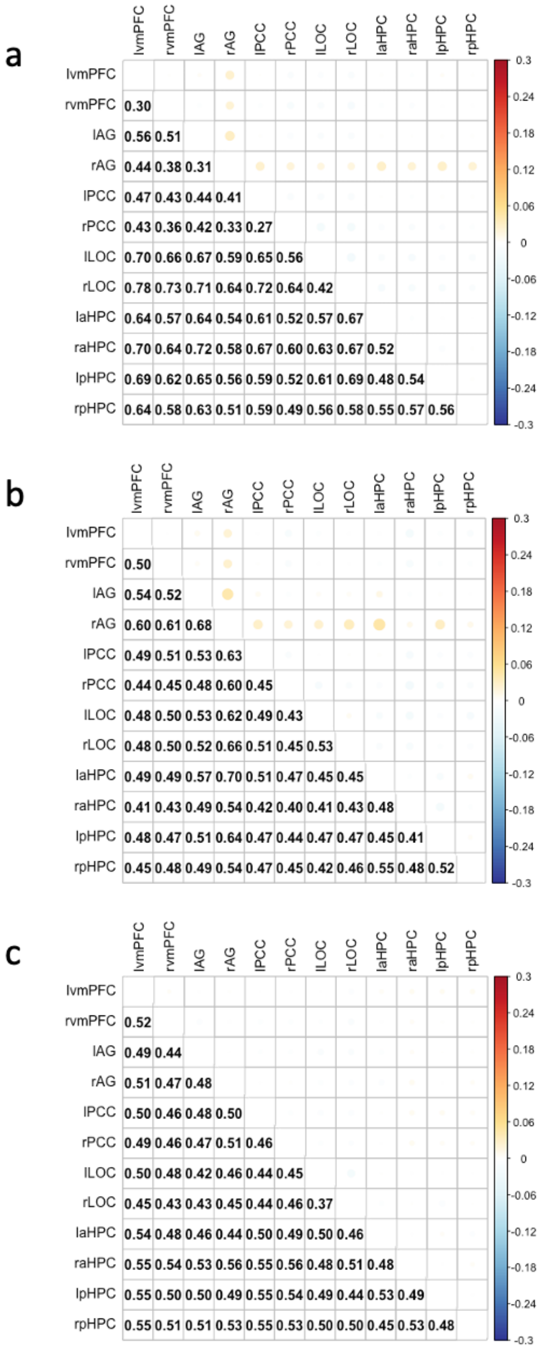

Figure S4. Effects of schema condition on post-encoding brain connectivity in all 23 participants. (A) Schema-C vs. Baseline, (B) Schema-IC vs. Baseline and (B) Schema-C vs. Schema-IC. The matrix shows region pair effects: the upper triangle illustrates the magnitude of the Fisher-transformed *z*-value, indicated by circle size and colour, and the lower triangle contains the P+ value.

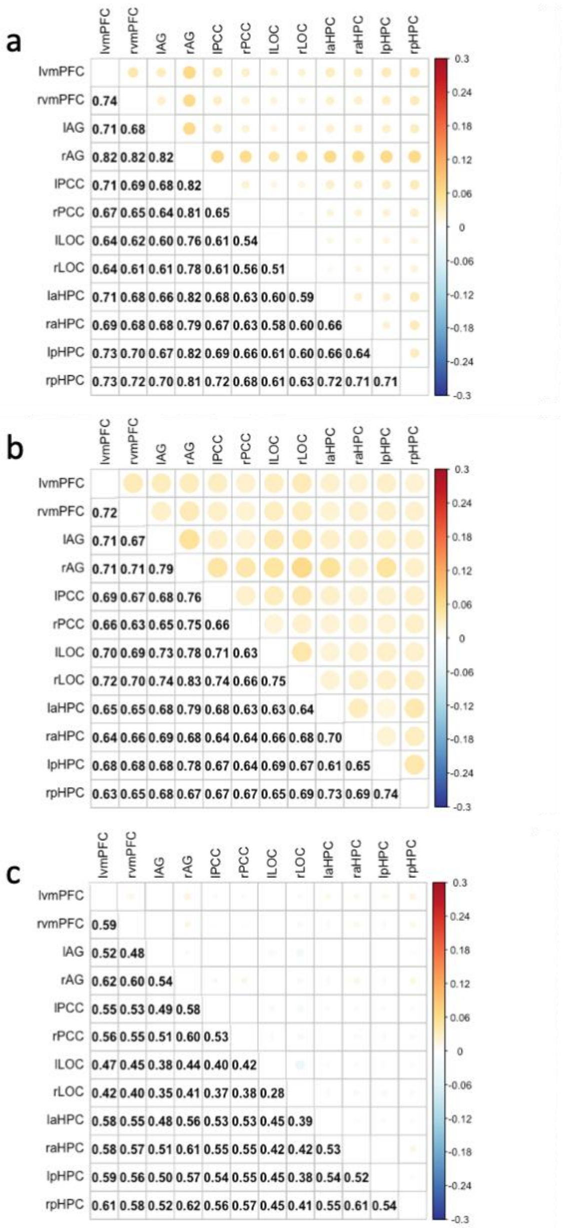

Figure S5. Effects of schema condition on post-encoding brain connectivity in 17 participants who demonstrated a schema effect. (A) Schema-C vs. Baseline, (B) Schema-IC vs. Baseline and (B) Schema-C vs. Schema-IC. The matrix shows region pair effects: the upper triangle illustrates the magnitude of the Fisher-transformed *z*-value, indicated by circle size and colour, and the lower triangle contains the P+ value.
